# Biodistribution and toxicity evaluation of oncolytic adenovirus Adf35(OGN) in Syrian hamster and mouse

**DOI:** 10.1038/s41417-025-00875-y

**Published:** 2025-02-11

**Authors:** Erik Yngve, Malin Eriksson, Anders Hedin, Arwa Ali, Chuan Jin, Olle Korsgren, Di Yu

**Affiliations:** 1 Department of immunology, genetics and pathology, Uppsala University, Uppsala, Sweden; 2 Department of Microbiology, Swedish Veterinary Agency, Uppsala, Sweden; 3 Department of Microbiology, Tumor and Cell Biology, Karolinska Institute, Stockholm, Sweden

**Keywords:** Immunization, Drug development, Cancer immunotherapy

## Abstract

Oncolytic adenovirus has been widely evaluated as a cancer treatment agent with tolerable toxicity profile. We have recently developed a new oncolytic adenovirus Adf35(OGN) with two immunostimulatory transgenes alpha-1,3-galactosyltransferase (*GGTA1*) from *Sus scrofa* and neutrophil-activating protein (NAP) from *Helicobacter pylori*. Adf35(OGN) can kill tumor cells and trigger a strong immune response against tumor antigens. Here, we report the toxicity and biodistribution of Adf35(OGN) in Syrian hamster and GGTA1-knockout mouse. The virus was delivered subcutaneously in naïve hamsters and intratumorally in *GGTA1-*knockout mouse in multiple doses at dosages of 1–5 × 10^11^ viral particles (VP)/kg. The virus did not replicate in any tissues, evidenced as low or no viral copies detected by qPCR. The virus was also found at low levels in biofluids (saliva, urine, and feces), indicating that spread to the environment is low with a low risk of secondary infections via shedding. The virus did not cause any biochemical, hematological, or histopathological alterations. In summary, Adf35(OGN) has a good safety profile in these animal models and these results support future clinical evaluation for Adf35(OGN).

## Introduction

Cancer immunotherapy has evolved rapidly over the last decades and is now established in many clinical treatment regimens. These therapies aim to boost the patient’s own anti-tumor immune response and overcome tumor immune escape mechanisms. Oncolytic viruses constitute a type of immunotherapy with potential to facilitate different steps in the anti-tumor immune response [1].

Mechanisms of action include the infection and lysing of tumor cells, leading to simultaneous release of tumor-associated antigens and pathogen- and damage-associated molecular patterns (PAMPs and DAMPs). This facilitates presentation of tumor-associated antigens in draining lymph nodes and subsequent priming and activation of tumor-reactive T-cells. To further improve the treatment efficacy, various transgenic immunostimulatory molecules expressed by the virus in infected target cells have been used to stimulate selected parts of the anti-tumor immune response [1].

At present, only one oncolytic virus, T-VEC (IMLYGIC), is approved in the United States and Europe for treatment of metastasized and unresectable malignant melanoma [2]. However, several clinical trials are completed or ongoing for various other oncolytic viruses for treatment of different types of cancer. Adenoviruses are commonly used as oncolytic viruses due to a favorable safety profile, with mild and transient side effects such as flu-like symptoms, headache, nausea, fatigue and dizziness, anemia, leuko- and thrombocytopenia, elevated liver transaminases, and diarrhea [3–5].

Adf35(OGN) is a new oncolytic adenovirus of serotype five with a fiber knob from serotype 35 to allow cell entry through CD46 which is a complement inhibitor which is upregulated in many types of cancer. A 24 bp deletion in gene E1A impair viral replication in normal cells but allows viral replication in tumor cells with dysregulated pRb-pathway [6]. The virus is equipped with two immunostimulatory transgenes, encoding alpha-1,3-galactosyltransferase and *Helicobacter pylori* neutrophil-activating protein (HP-NAP), creating a principally new pathway to provoke anti-tumor immunity.

Alpha-1,3-galactosyltransferase, encoded by *GGTA1*, is an enzyme that synthesizes galactose-alpha-1,3-galactose (α-gal), a common glycosylation structure in mammals and bacteria. Due to a mutation in *GGTA1*, humans lack expression of α-gal and instead anti-α-gal antibodies naturally occur in substantial titers, making α-gal one of the most potent immune activators known in humans [7–9].

HP-NAP is a virulence factor of *Helicobacter pylori* and is known to attract neutrophils and monocytes and activate them to produce Th1-polarizing cytokines [10, 11], which may promote and maintain Th1-polarized anti-tumor T-cell responses.

Through its ability to infect and lyse tumor cells and express α-gal and NAP, Adf35(OGN) may have the ability to revert the immunosuppressive tumor microenvironment, seen in many solid tumor types. The treatment may further increase immunosurveillance for tumor-specific neoantigens and ultimately stimulate expansion of cytotoxic tumor-reactive T cells, that specifically kill tumor cells, both in the treated tumor and also in non-injected lesions.

Pre-clinical safety studies are needed before new oncolytic adenoviruses enter clinical trials. Human adenoviruses are species-specific and both cellular entry and replication are limited in murine cells [12], while replication in Syrian hamster cell lines have been nearly as effective as in human cell lines [13, 14]. Therefore, Syrian hamster, also known as Golden hamster, is the best available rodent model and it is commonly used for kinetic, biodistribution, and toxicity studies of oncolytic adenoviruses.

The natural expression of α-gal in Syrian hamster cells disables this model to evaluate the toxicity of virally induced α-gal expression. This part of the treatment can only be tested fully in humans or, with limitations, in *GGTA1*-knockout mouse. This mouse lacks expression of α-gal and, thus, can be immunized against the antigen in order to mimic the human situation with preexisting anti-α-gal antibodies.

Here, we investigated the biodistribution and toxicity of Adf35(OGN) in Syrian hamster. Histopathological toxicity was additionally studied in *GGTA1*-knockout mouse, to discover the effect of transgenic α-gal expression.

The treatment was well tolerated and without significant effect on blood biochemistry, hematology, or histopathology. There were no signs of viral replication in tissues and shedding was sparse. These results support a phase Ia dose-escalation clinical trial for Adf35(OGN).

## Material and methods

### Viruses

Adf35(OGN) has a backbone from wild-type adenovirus serotype 5. To facilitate entry into tumor cells, the CAR-binding fiber knob of serotype 5 is replaced by the fiber knob of serotype 35, which allows viral entry through interaction with CD46, a cell surface protein abundantly expressed on many tumor types.

A 24 bp-deletion of the *E1A* and full deletion of the *E1B* gene prevent viral replication in normally functioning cells while allowing replication in tumor cells with aberrant p53 and Rb-E2F mediated cell cycle control.

The *E1B*-region is replaced by a sequence encoding alpha-1,3-galactosyltransferase and HP-NAP.

This study was performed to test a batch of Adf35(OGN) that was produced under GMP standards and is intended for clinical use. However, due to limited availability of the GMP grade virus, a batch of laboratory grade Adf35(OGN) was used for some groups in the study (groups 9 and 10), to test an intensified treatment schedule with three instead of two repeated injections.

Adf35(Mock) was used as a non-replicating control. Its surface structure is identical to Adf35(OGN). It has a complete deletion of the E1A and E1B genes, inhibiting any viral replication, and does not carry any transgenes.

For the mouse experiment Adf35(OGN), and three different variations of Adf35(OGN) were used: Adf35(O) lacking transgenes, Adf35(OG) with α-gal only, and Adf35(ON) with HP-NAP only. Non-replicating adenoviral vector Adf35(Luc), expressing the transgene luciferase was used as a negative control.

### Ethics statement

All experimental procedures on the hamsters and mice were approved by the regional animal ethics committee of Uppsala, Sweden (Dnr. 5.8.18-08027/2021 and C164/15 with renewal 5.8.18-006672/2021) and performed in accordance with the EU directive 2010/63/EU on the protection of animals used for scientific purposes.

### Hamster

Seven-week-old male and female Syrian/Golden hamsters RjHan: AURA were purchased from Janvier-Labs and housed at the Swedish Veterinary Agency, Uppsala, Sweden. The hamsters were single-housed in Sealsafe GR900 (Tecniplast; Buguggiate, Italy) on aspen bedding (Tapvei, Harjumaa, Estonia) under controlled climate conditions with a 12 h light, 12 h dark cycle and monitored at least once daily. The cages were enriched with bedding material, polycarbonate houses, chewing sticks, and sand baths. The hamsters had unlimited access to drinking water and were fed *ad lib* with Nature Hamster (Versele-Laga, Deinze, Belgium), Complete Hamster & Gerbil (Versele-Laga, Deinze, Belgium), and Tiny Friends Farm Harry Hamster tasty mix (Supreme Petfoods, Ipswich, United Kingdom). During the 7-days acclimatization, the hamsters were trained to be accustomed to the handler and prepared for the experimental procedures.

**Fig. 1:**
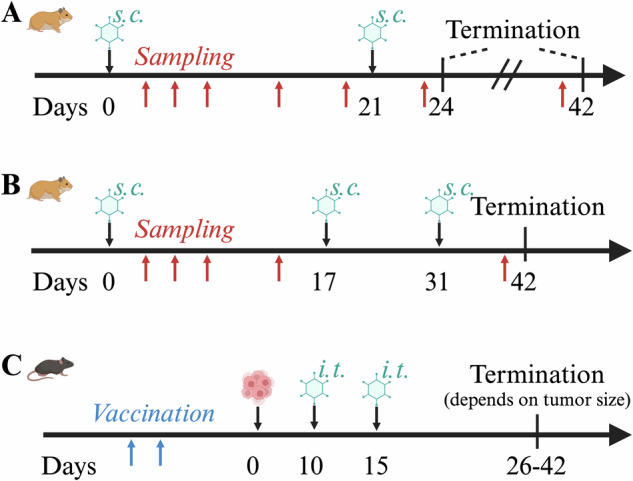
Treatment schedule for hamster and mouse experiments. Hamsters were treated either by two (**A**) or three (**B**) subcutaneous (s.c.) injections, followed by either early (day 24) or late (day 42) termination. *GGTA1*-KO mice (**C**) were vaccinated against a-gal followed by tumor implantation. When palpable tumor established, mice were treated by two intratumoral (i.t.) injections and termination was performed when tumor size exceeded 1000 mm^3^, defined as the humane endpoint criterium. Created in BioRender.

Treatment scheme, doses, and route of administration are presented in Fig. 1 (detailed in Table S1). The hamsters were randomly assigned to treatment groups without formal protocol, with three males and three females per group receiving two subcutaneous injections on days 0 and 21. They were injected with either PBS, Adf35(Mock), or GMP grade Adf35(OGN) at a dose of either 1 × 10^11^ or 5 × 10^11^ VP/kg. At day 24 three hamsters per group were euthanized (later referred to as “early termination”) and the remaining animals were euthanized at day 42 (“late termination”). A group of two males and two females (group 9) were treated with laboratory grade Adf35(OGN) in an intensified treatment schedule with three subcutaneous injections at day 1, 17, and 31 followed by termination at day 34. As a control, two animals (group 10) were treated with laboratory grade Adf35(OGN) according to the standard schedule with two injections followed by early termination. Two males and two females were left untreated as negative controls (group 11) and were euthanized at day 17. This experiment has not been repeated.

### *GGTA1*-knockout mouse

*GGTA1*-knockout mouse on a C57BL/6 background [15] was a gift from Dr. Park’s lab (Seoul National University), originated from Dr. D’Apice’s lab (St. Vincent’s Hospital), and was kept as in-bread at the animal facility of Rudbeck Laboratory, Uppsala University, Sweden. They were housed in groups of 3–5 individuals in single ventilated cages under controlled climate conditions with a 12 h light, 12 h dark cycle and monitored at least once daily. The cages were enriched with bedding material, polycarbonate houses and paper wool, with free access to food and water.

Mouse *GGTA1*-knockout pancreatic ductal adenocarcinoma cells (Panc02-KO) (cell line not authenticated) were cultured in DMEM (Dulbecco’s Modified Eagle Medium) complemented with 1% sodium pyruvate, 1% Pesticides (Penicillin (100 U/ml) and Streptomycin (100 g/ml)) and 10% fetal bovine serum. Media and supplements were from Invitrogen. Mycoplasma was routinely tested during cell culture.

Mice were immunized against α-gal by two injections of mucin and polyinosinic-polycytidylic acid (poly I: C) whereafter Panc02-KO cells were injected subcutaneously into the right flank. When palpable tumors were formed, mice were assigned randomly to treatment groups without formal protocol. Two mice per group were injected with PBS or 1 × 10^11^ VP per mouse of Adf35(Mock), Adf35(O), Adf35(OG), Adf35(ON), or Adf35(OGN) on day 10 and 15, according to Fig. 1 (detailed in Table S2). When tumor size reached 1000 mm^3^, the humane endpoint of the experiment, mice were euthanized and organs were collected. Termination dates ranged from 11 to 29 days after last viral injection. Heart, lung, spleen, liver, kidney, ventricle, and brain (except three animals) were collected and fixed in 4% buffered formaldehyde for histopathological analysis. This experiment has not been repeated.

### Sampling

From the hamsters, biofluids including feces, urine-soaked bedding and/or sand, and buccal swabs were collected on day 1, 3, 6, 12, and 21 after the first treatment and at termination. The cages, including the interior materials and food, were changed on the day before the sampling to capture the actual shedding at the sampling time-point.

At termination, the hamsters were anesthetized with isoflurane for collection of blood through heart puncture followed by decapitation. Tissue samples of ~2 × 2 × 2 mm for biodistribution analysis were collected from brain, spinal cord, heart, lungs, mammary gland/skin, ventricle, small intestine, large intestine, liver, pancreas, femoral bone marrow, adrenal gland, mesenteric lymph node, testicle, epididymis, prostate, ovaries, uterus, and urinary bladder. Mammary gland tissue was difficult to isolate during necropsy and these biopsies represent a combination of mammary gland tissue, skin, and subcutaneous tissue. To minimize contamination between tissue samples, the instruments were sterilized in a glass bead sterilizer (Hot bead sterilizer—FST 250, Fine Science Tools, Foster City, CA, USA) according to manufacturer’s instructions. Biofluids including feces, saliva, and urine were also collected. In case the urinary bladder contained urine a sample was taken by cystocentesis, otherwise urine was collected from the bedding and/or sand. All samples intended for biodistribution analysis were stored at −80 °C until analysis. The remaining tissue from the respective organs and in addition the sternum and optic nerve were fixed in 4% buffered formaldehyde or in Davidson’s solution and later used for histopathological analysis.

### Anti-adenovirus IgG titration

To monitor the humoral immune response against Adf35(OGN), anti-adenovirus IgG was measured by ELISA on serum collected at necropsy.

Polystyrene high bind ELISA plates (Costar 3361) were coated with viral particles through the incubation of 4 × 10^11^ VP/mL Adf35(OGN) in PBS at 4 °C overnight. Two percent BSA diluted in PBS was used for blocking and incubated for 2 h. Hamster serum collected at necropsy and diluted 1:1000 in PBS was applied and incubated for 1 h at room temperature. Secondary antibody, goat anti-Syrian hamster IgG, conjugated with horse reddish peroxidase (HRP), (Abcam 6892) diluted 1:8000 was applied and incubated for 1 h at room temperature. Substrate (Thermo Scientific cat. 34028) was applied for HRP detection and the signal was analyzed at 450 nm (BioRad Imark Microplate reader).

### Blood analyses

Whole blood was collected in EDTA-tubes at necropsy and hemoglobin, erythrocyte volume fraction, leukocyte, thrombocyte, neutrophil, eosinophil, lymphocyte, and monocyte counts were analyzed within 8 h from necropsy. Serum was extracted from untreated blood, following 10 min centrifugation at 400 × *g*. Serum analyses include alanine aminotransferase (ALT), aspartate aminotransferase (AST), alkaline phosphatase (ALP), total protein, albumin, and creatinine. All analyses were performed at the Clinical Chemical Laboratory at the University Animal Hospital, Uppsala, Sweden. There is limited experience of blood hematology and biochemistry in hamster and reference values are lacking. Therefore, results were compared to internal controls including untreated and PBS treated animals.

### Biodistribution

qPCR was used to detect and quantify viral genome copies in tissues and biofluids. The analysis was performed under GLP standard at TATAA Biocenter, Gothenburg, Sweden. DNA extraction form tissues and biofluids was optimized and validated. Tissue pieces of ~2 × 2 × 2 mm were used for input and DNA was isolated and eluted in 100 µL water. A qPCR-based assay detecting the viral gene E4orf1 and vertebrate genomic DNA was optimized and validated. Two microliters isolated DNA was used as template in each reaction. The assay limit of detection (LOD) was defined as the last concentration, in a 2× incremental dilution series ranging from 8000 to 0.244 copies per reaction, where at least 95% of the reactions detected the presence of the template DNA, i.e., less than 5% false negatives. Limit of quantification (LOQ) was defined as the last point where the relative standard deviation of the replicates in the standard curve was <35%. LOD and LOQ were determined to 20 VP/reaction, which approximately correlates to 1000 VP per sample. Isolated DNA was analyzed in technical duplicates. Samples were included in further analyses if at least one replicate had a viral particle number above 20. If no signal was detected in the other replicate, the viral particle number of the sample was set to the number of the replicate where virus was detected above LOD. If viral particles were detected (below or above LOD) in both samples, the average was used for the further analysis.

### Histopathology

Histopathology was used to examine treatment-related toxicity in various tissues, collected at necropsy. Biopsies were fixed and stored in 4% formaldehyde until further processing. The optic nerves, testes, and epididymides were fixed in Davidsons’s solution for 48 h and then transferred to 4% buffered formaldehyde. The preparation and examination of tissues were performed unblinded under GLP-standard by BioVet Veterinary Medical Laboratory, Sollentuna, Sweden. Tissues were paraffin-embedded, sectioned (4–6 µm), stained with hematoxylin-eosin, and examined by light microscopy.

### Statistics

Data was analyzed using R (v. 4.3.0) in Rstudio (v. 2023.6.2.561) [16].

Non-parametric tests were used to assess differences between groups. Kruskal–Wallis test was used to detect differences between groups for each assayed hematological and biochemical parameter as well as anti-adenovirus titer. Any significant deviation from the expected values would be further assessed using post-hoc Dunn’s test between any pair of groups, using Bonferroni- adjusted *p*-values to correct for multiple testing.

To increase power, the groups were also pooled into a Control group (Groups 1,2 and 11) and a Treatment group (Groups 3–10) which were then tested using Mann–Whitney *U*-test for each hematological and biochemical parameter as well as the anti-adenovirus titer. In all cases, differences with *p* ≤ 0.05 were considered significant.

For bio-distribution and shedding data no statistical analyses were performed.

## Results

### Adf35(OGN) does not cause observable adverse effects

Syrian hamster and *GGTA1*-knockout mouse were treated with repeated injections of virus according to Fig. 1. Throughout the experiment no adverse effects or signs of discomfort were observed by visual inspection, indicating that treatment with Adf35(OGN) is well tolerated.

### Adf35(OGN) does not replicate in major organs

**Fig. 2:**
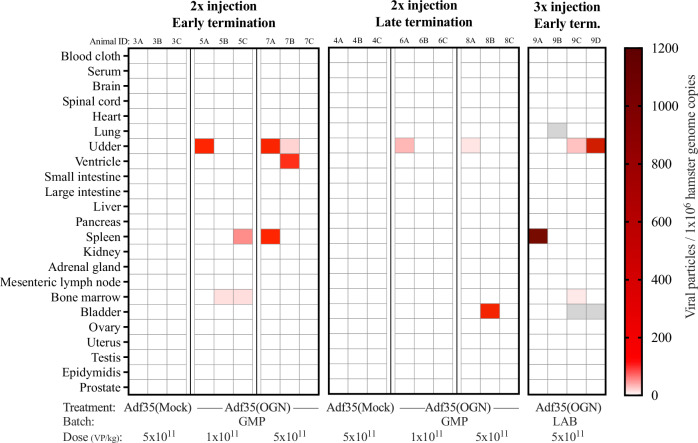
Viral biodistribution in tissues after Adf35(OGN) treatment. Hamsters were injected subcutaneously with Adf35(Mock) and Adf35(OGN) and samples were harvested at either early or late termination points (left and middle columns). Animals injected three times are shown in the right column. Tissue type and treatment is indicated on the *y*-axis and *x*-axis respectively. Adenovirus and hamster DNA was quantified by qPCR and normalized viral particle numbers (against hamster DNA) are shown for each individual sample, as indicated by the color legend. White fill indicates that virus was not detected above LOD in the sample. Gray fill indicates analysis error. GMP: GMP grade, LAB: laboratory grade, Term.: termination.

To study the tissue distribution and replication of Adf35(OGN), samples were analyzed by qPCR for detection and quantification of viral particles. The results from individual samples are shown in Fig. 2 and detailed in Table S3. Viral particles above LOD were found in only 15 of 451 samples, collected from animals treated with virus Adf35(Mock) or Adf35(OGN), indicating limited viral spread and replication. The positive samples were found in mammary glands/skin, ventricle, spleen, bone marrow, and bladder. The viral numbers ranged from 12 to 1 170 VP per 1 × 10^6^ hamster genome copies (Table S3).

No samples above LOD were found in the PBS and Adf35(Mock) groups (Fig. 2). In the Adf35(OGN) treatment group, five samples were positive in the low dose group (*n* = 6), six were positive in the corresponding high dose group (*n* = 6) and four were positive in the group treated with high dose and three injections (*n* = 4) (Fig. 2). As expected, more samples were positive in animals treated with the high dose compared to the low, but no difference was noted between two and three injections. Out of eleven positive samples in the groups comparing different termination timepoints, eight were found in the early termination and three were found at the late termination (Fig. 2). This indicates that viral particles gradually were cleared from the tissues.

In summary, viral particles were mostly not detected or detected in low copy numbers, indicating that local injection of Adf35(OGN) causes limited systemic spread without significant replication in the investigated tissues. This is likely due to slow release of viral particles into the systemic circulation, the mutated viral *E1*-gene, and the anti-viral antibodies. These findings suggest a low risk of adverse effects from Adf35(OGN) therapy.

### Adf35(OGN) does not cause significant viral shedding

**Fig. 3:**
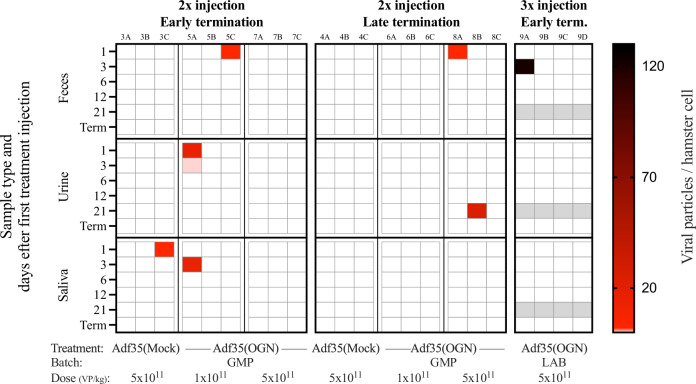
Viral shedding in biofluids after Adf35(OGN) treatment. Naive hamsters were injected subcutaneously with Adf35(Mock) or Adf35(OGN) and biofluids including feces, urine, and saliva were collected. Sample type and timepoint of collection are indicated on the left *y*-axis and treatment is indicated in the bottom *x*-axis. Adenovirus DNA was quantified by qPCR and normalized viral particle numbers (Viral particles per hamster cell) are shown for each individual sample, as indicated by the color legend. Gray fill indicates that samples were not collected. GMP: GMP grade, LAB: laboratory grade, Term.: termination.

To study viral shedding into the surrounding environment, biofluids (feces, urine, and saliva) were collected and analyzed after local delivery of Adf35(OGN). Viral particles were detected in eight out of 384 samples (Fig. 3). Seven of these samples were collected 1 or 3 days after the injection. Only one positive sample was found among the samples collected day 21. In almost all cases, the positive samples were retrieved from different animals, and in only one individual, viruses were found in both urine and saliva. Positive samples were more common in animals treated with Adf35(OGN) than Adf35(Mock) (Fig. 3). The viral numbers detected were generally low and maximally 121 VP per hamster genome copy (Table S3). Taken together, these results show that repeated local delivery of Adf35(OGN) does not result in any significant viral shedding.

### Adf35(OGN) stimulates a humoral anti-viral response

**Fig. 4:**
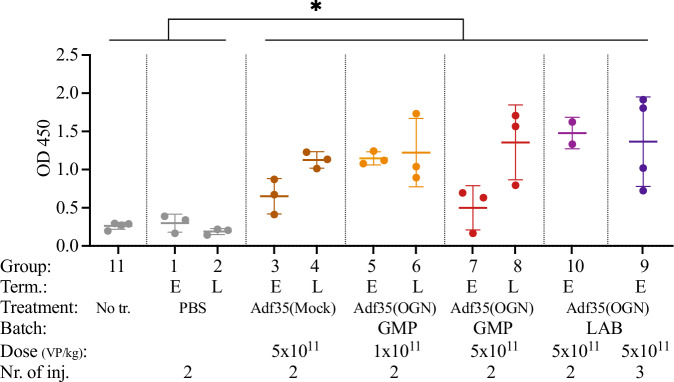
Anti-adenovirus IgG levels in hamsters after Adf35(OGN) treatment. Hamsters were injected subcutaneously with Adf35(Mock) and Adf35(OGN). Untreated and PBS-injected hamsters served as controls. Anti-adenovirus IgG were measured by ELISA on samples harvested at either early (E) or late (L) termination points as indicated. Individual values from each animal, as well as mean ± SD are shown. Mann–Whitney U test was performed to compare virus treated animals (pooled) to the control group (pooled untreated and PBS-injected animals). *p < 0.05. GMP: GMP grade, LAB: laboratory grade, Term.: termination.

In general, the viral injections stimulated production of anti-adenovirus IgG antibodies (*p* = 1.5x${10}^{-6}$) (Fig. 4), that were detected in the early termination groups and were maintained also in the late termination groups. The tested viruses (Adf35(Mock) and Adf35(OGN)), doses (1 × 10^11^ and 5 × 10^11^ VP/kg), and numbers of injections (two or three) all resulted in similar IgG levels (Fig. 4). This indicates that treatment with Adf35(OGN) stimulates a humoral immune response.

### Adf35(OGN) does not affect blood hematology and biochemistry

**Fig. 5:**
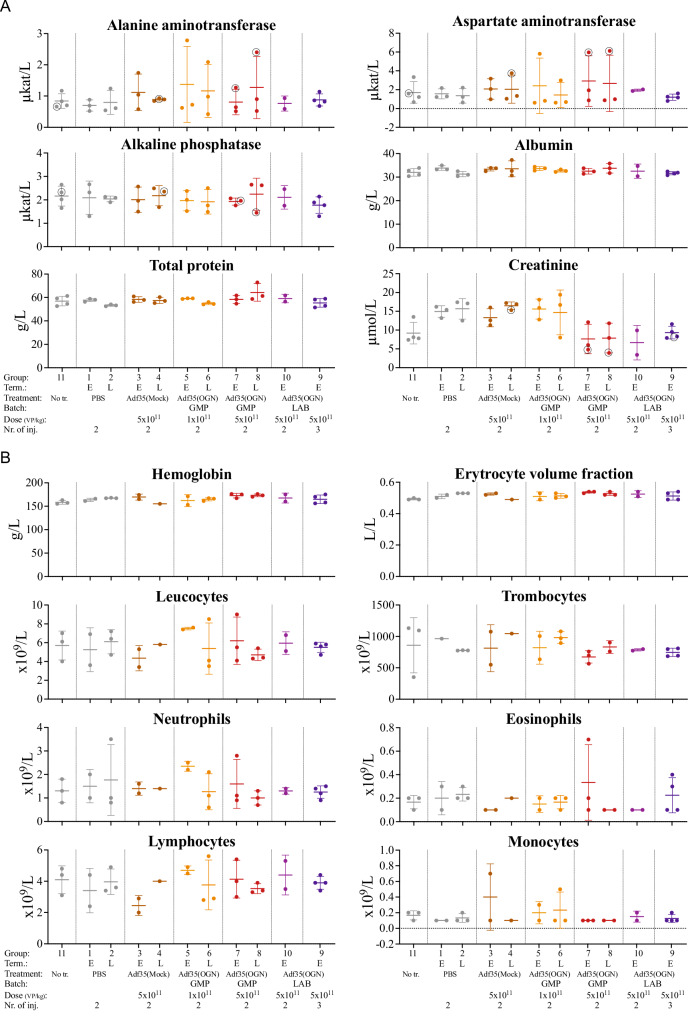
Serum biochemistry and hematology in hamsters after Adf35(OGN) treatment. Hamsters were injected subcutaneously with Adf35(Mock) and Adf35(OGN). Untreated and PBS-injected hamsters served as controls. Analyses including biochemistry (**A**) and hematology (**B**) were performed on samples harvested at either early (E) or late (L) termination points as indicated. Individual values from each animal, as well as mean ± SD are shown. Mann–Whitney U and Kruskal–Wallis tests were used to identify any statistically significant differences between virus treated groups and controls. Individuals with hemolysis index above three are marked with a black circle where relevant. GMP: GMP grade, LAB: laboratory grade.

Blood was collected at termination and common blood analyses were performed. Results from the respective treatment groups were compared to the internal controls of untreated and PBS-injected animals (Fig. 5A, B), applying Mann–Whitney U and Kruskal–Wallis tests. None of the analyzed parameters showed any statistically significant difference between virus treated groups and controls, when analyzing both pooled and individual virus treated groups. Some individual values from virus treated groups (mainly ALT and AST) were outside the range of the control group but in many cases these samples were affected by hemolysis. Overall, the results indicate that the given treatments have little effect on hematological and biochemical homeostasis.

### Adf35(OGN) does not cause histopathological alterations in hamster

**Fig. 6:**
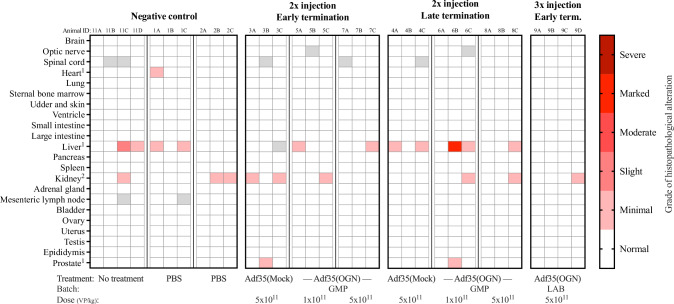
Histopathological alterations in hamster tissues after Adf35(OGN) treatment. Hamsters were injected subcutaneously with Adf35(Mock) and Adf35(OGN) and samples were harvested at either early or late termination points (two middle columns). Animals injected three times are shown in the right column. Untreated and PBS-injected hamsters served as controls and are shown in the left column. Tissue type and treatment is indicated on the *y*-axis and *x*-axis respectively. Histopathological examination was performed and grade of histopathological alterations, ranging from minimal to severe, is indicated by the color legend. Gray fills indicate that samples are missing or that the structure of interest could not be identified in the collected material. ^1^: Inflammatory cell infiltrates, mainly lymphocytes, macrophages, and plasma cells. ^2^: Papillary or cortical kidney mineralization. GMP: GMP grade, LAB: laboratory grade, term.: termination.

A histopathological analysis was performed to identify if treatment with Adf35(OGN) induced tissue lesions in hamsters. Liver biopsies (11 out of 34) were found to have periportal or lobular infiltrates of mononuclear cells, mainly lymphocytes, scattered macrophages, and plasma cells (Fig. 6). Since these alterations were found across all groups including untreated animals, treatment or dose correlation could not be confirmed, and the alteration was regarded incidental. Discrete immune cell infiltrates were occasionally observed in heart and prostate and papillary kidney mineralization was seen in 12 out of 34 animals across all treatment groups. None of these findings were considered related to the treatment. No alterations were observed in the brain, optic nerve, ventricle, intestines, lungs, pancreas, sternum, lymph node, adrenal gland, spleen, mammary gland, bladder, ovary, uterus, testes, or epididymides (Fig. 6).

In summary, microscopic alterations were identified in some organs, but with even distribution in both treated groups and controls, thus none of these alterations were considered treatment-related.

### Adf35(OGN) does not cause histopathological alterations in GGTA1-knockout mouse

**Fig. 7:**
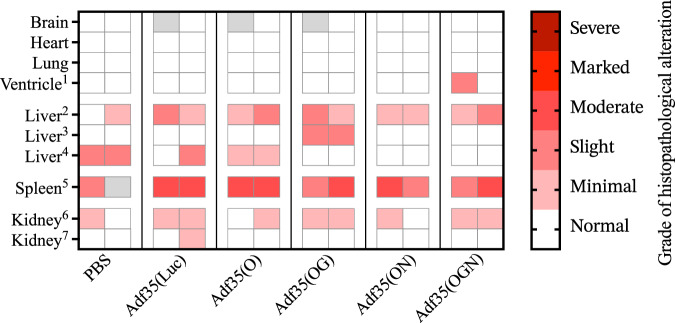
Histopathological alterations in *GGTA1*-knock out mouse after Adf35(OGN) treatment. *GGTA1*-knock out mice were injected intratumorally with PBS, Adf35(Luc), Adf35(O), Adf35(OG), Adf35(ON) or Adf35(OGN). Samples were harvested when tumors size reached the humane end-point. Tissue type and treatment are indicated on the *y*-axis and *x*-axis respectively. Histopathological examination was performed and grade of histopathological alterations, ranging from minimal to severe, is indicated by the color legend. Gray fills indicate that samples are missing or that the structure of interest could not be identified in the collected material. ^1^: Inflammatory cell infiltrates, mainly granulocytes (neutrophilic and eosinophilic) with few mononuclear cells (lymphocytes and macrophages).^2^: Inflammatory cell infiltrates in liver parenchyma, mainly lymphocytes, macrophages, and neutrophilic granulocytes. ^3^: Inflammatory cell infiltrates in centrilobular areas of the liver, mainly lymphocytes and macrophages. ^4^: Multifocal hepatocellular hypertrophy in the centrilobular areas of the liver. ^5^: Extramedullary hematopoiesis in the spleen. ^6^: Tubular dilatation in the renal cortex. ^7^: Papillary renal mineralization.

In the *GGTA1*-knockout mouse model, animals were treated with PBS, Ad35(Luc), Adf35(O), Adf35(OG), Adf35(ON) and Adf35(OGN) respectively (*n* = 2 in each group), after immunization to generate anti-α-gal antibodies and tumor implantation (Fig. 1). No microscopic alterations were observed in the brain, heart, and lung (Fig. 7). In the ventricle, slight infiltration of inflammatory cells, mainly granulocytes, were observed in the lamina propria of one animal treated with Adf35(OGN). In the liver, 11 out of 12 animals had minimal to slight infiltration of inflammatory cells, whereas centrilobular perivascular infiltration was specifically observed in animals treated with Adf35(OG) (Fig. 7). 5 out of 12 animals were found to have minimal to slight centrilobular hypertrophy in PBS, Adf35(Luc) and Adf35(O) treated groups (Fig. 7). In the spleen, slight to moderate diffuse extra medullary hematopoiesis were observed in all animals. In kidneys, minimal multifocal tubular dilatation in cortex was observed in 9 out of 12 animals across all treatment groups (Fig. 7).

In summary, *GGTA1*-knockout mice treated with *GGTA1*-expressing viruses, Adf35(OG) and Adf35(OGN), showed a similar frequency and severity (minimal or slight) of histopathological alterations as those treated with control viruses or PBS. These alterations were most likely caused by the immunization and tumor implantation rather than the viral treatment.

## Discussion

This preclinical safety, toxicity, and biodistribution study shows a favorable safety profile of Adf35(OGN). It is supported by low tissue replication, low environmental shedding, maintained blood homeostasis, and no treatment-related histopathological alterations.

The biodistribution of Adf35(OGN) was assessed by measuring the levels of viral DNA in various tissues and biofluids. The results showed that the levels of viral DNA were low or undetectable in all examined organs. Only 15 out of 451 tissue samples and 8 out of 420 biofluid samples were found positive for viral particles, and blood cloth, serum, and liver were all negative. This indicates that the virus has a limited systemic spread, which is likely due to the local subcutaneous injection. This is also in line with a previous study indicating that distribution of virus injected subcutaneously is limited in comparison to virus injected intravenously [17]. In the tissue sample with the highest level of viral DNA the ratio of viral genome copies to hamster genome copies were still very low, 0.002 VP per hamster cell. This indicates that no significant viral accumulation or replication occurs in any of the investigated tissues. Moreover, the already low levels of viral DNA in the early termination group further declined in the late termination group, indicating that viral particles entering systemic circulation were effectively cleared by antiviral antibodies, which could be detected in the early termination groups and were maintained over time. In addition, low levels of viral DNA in feces, urine, and saliva, suggest that the spread of viral particles to the environment is low, and secondary infections are unlikely.

Histopathological examination revealed no tissue alterations related to the treatment. Aberrant findings were in all cases regarded as common features of the model or incidental. This interpretation is further supported by normal biochemistry, hematology, and low levels of viruses in aberrant samples. This is in line with previous studies that have found a safe toxicity profile of oncolytic adenoviruses injected subcutaneously [17] and by other routes [18].

Analysis of common biochemistry and hematology parameters did not show any statistically significant difference between treated groups and controls, even when virus-treated groups were pooled to increase power. Bearing in mind the low number of animals in the study and thus low statistical power and limited ability to show differences between treated groups and controls, the results indicate that local delivery of Adf35(OGN) does not affect biochemistry or hematology in any significant way.

Increased levels of ALT and AST, as previously described in response to adenoviral treatment [18–20], were occasionally observed also in this study, although hemolysis may also have contributed to the increase in some of the cases. These alterations were not accompanied by more severe or frequent hepatic histopathological alterations, and thus, the biological relevance of the increase in ALT and AST levels is regarded low.

The intensified treatment schedule with three injections instead of two did not change the overall picture for any of the analyses including biodistribution, biochemistry, hematology, and histopathology. This indicates that repeated doses are well tolerated.

Human adenovirus does not replicate in mouse [12–14], neither in Panc02 cells that were used for tumor implantation [12]. Yet, we consider the *GGTA1*-knockout mouse still to be the best available model to study the toxicity related to transgene, and expression of α-gal. In this mouse study, independently evaluated by an experienced animal pathologist, minimal to slight histopathological alterations were seen, mainly in liver, spleen, and kidney, in all groups of animals treated with PBS, non-replicating Adf35(Luc) or replicating viruses with transgene expression. This is likely due to the extensive interventions during model establishment, including immunization against α-gal and tumor cell implantation. We, therefore, conclude that repeated local intratumoral injections of Adf35(OGN) did not cause any lesions and alterations in major organs, indicating a favorable safety profile of the virus. However, a complete safety assessment of Adf35(OGN) can only be performed on data from human clinical trials, in which viral infectivity and replication are uncompromised and the immune activation, caused by transgene expression, is fully representative.

In conclusion, this study indicates that repeated doses of Adf35(OGN) are safe in a dose range between 1 × 10^11^ and 5 × 10^11^ VP/kg in Syrian hamster. The results support a clinical dose escalation study with repeated intratumoral injections as a cancer treatment.

## Supplementary information

Supplemental Tables 1–3

## Data Availability

Data is accessible upon a sensible inquiry.
