# Supplementary material for: Biodistribution and toxicity evaluation of oncolytic adenovirus Adf35(OGN) in Syrian hamster and mouse
Source: Cancer Gene Ther. 2025 Feb 11;32(3):297–305. doi: 10.1038/s41417-025-00875-y (PMC11946883; doi:10.1038/s41417-025-00875-y)
Supplement: Supplementary file 1 — Supplemental Tables 1–3 [file 41417_2025_875_MOESM1_ESM.pdf]

Supplementary table 1. Overview of animals, groups and procedures in the toxicity and biodistribution study in Syrian hamster.

Group (no.)	Treatment (s.c.)	Dose (VP/kg)	Timepoints (days[1])		Sex[2]	Animal ID
			Treatment	Termination		
1	PBS	100 uL PBS buffer No virus	0, 21	24	F	1A
					F	1B
					M	1C
2				42	F	2A
					M	2B
					M	2C
3	Adf35(Mock)	$5x10^{11}$		24	F	3A
					M	3B
					M	3C
4				42	F	4A
					F	4B
					M	4C
5	Adf35(OGN) GMP	$1x10^{11}$		24	F	5A
					F	5B
					M	5C
6				42	F	6A
					M	6B
					M	6C
7	Adf35(OGN) GMP	$5x10^{11}$		24	F	7A
					M	7B
					M	7C
8				42	F	8A
					F	8B
					M	8C
9	Adf35(OGN) LAB	$5x10^{11}$	0, 17, 31	24	M	9A
					M	9B
					F	9C
					M	9D
10			0, 21		F	10A
					F	10B
11	No treatment	N/A	N/A	17	F	11A
					F	11B
					M	11C
					M	11D

[1] Days counted from first treatment injection. [2] F = Female; M = male.

Supplementary table 2. Overview of animals, groups and procedures in the toxicity study in *GGTA1*-knock out mouse.

Group (no.)	Treatment (s.c.)	Dose (VP/mouse)	Timepoints (days*)		Animal ID
			Treatment	Termination	
1	PBS	N/A	10, 15	28	E:00012 R
				37	E:00012 L
2	Adf35(Luc)	$1x10^{11}$		26	E:00010 N
				37	E:00011 N
3	Adf35(O)			26	E:00014 N
				44	E:00014 L
4	Adf35(OG)			26	E:00023 R
				44	E:00023 L
5	Adf35(ON)			37	E:00022 L
				44	E:00022 B
6	Adf35(OGN) LAB			28	E:0003 R
				30	E:0005 R

* Days counted after tumor implantation, which is designated as day 0.

Supplementary table 3. Data summary in the hamster study, including individual values for tissue biodistribution, shedding, histopathology, hematology and biochemistry.

Group	1			2			3			4			5			6			7			8			9				10		11			
Animal ID	1A	1B	1C	2A	2B	2C	3A	3B	3C	4A	4B	4C	5A	5B	5C	6A	6B	6C	7A	7B	7C	8A	8B	8C	9A	9B	9C	9D	10A	10B	11A	11B	11C	11D
Sex	F	F	M	F	M	M	F	M	M	F	F	M	F	F	M	F	M	M	F	M	M	F	F	M	F	M	F	F	M	M	F	F	M	M
Treatment _batch	PBS						Adf35(Mock)						Adf35(OGN)_GMP						Adf35(OGN)_GMP						Adf35(OGN)_LAB						No treatment			
Dose (VP/kg body weight)							5,00E+11						1,00E+11						5,00E+11						5,00E+11									
Treatment days	0 and 21																								0, 17 and 31				0 and 21					
Termination day	24			42			24			42			24			42			24			42			34				24		17			
Biodistribution in tissues (VP/1x10^6 hamter genome copies)																																		
Blood cloth																																		
Serum																																		
Brain																																		
Spinal cord																														35,6				
Heart																																		
Lung																																		
Udder													146,2			40,1			179,2	25,2		14,8					35,2	514,4		87,9				
Ventricle																				110,5														
Small intestine																																		
Large intestine																																		
Liver																																		
Pancreas																																		
Spleen															63,0				144,1						1170									
Kidney																																		
Adrenal gland																																		
Mesenteric lymph node																																		
Bone marrow														17,7	18,8												12,4							
Bladder																							219,2											
Ovary			N/A		N/A	N/A		N/A	N/A			N/A			N/A		N/A	N/A		N/A	N/A			N/A			N/A	N/A		N/A				
Uterus			N/A		N/A	N/A		N/A	N/A			N/A			N/A		N/A	N/A		N/A	N/A			N/A			N/A	N/A		N/A				
Testis	N/A	N/A		N/A			N/A			N/A	N/A		N/A	N/A		N/A			N/A			N/A	N/A		N/A	N/A			N/A					
Epididymis	N/A	N/A		N/A			N/A			N/A	N/A		N/A	N/A		N/A			N/A			N/A	N/A		N/A	N/A			N/A					
Prostate	N/A	N/A		N/A			N/A			N/A	N/A		N/A	N/A		N/A	N/A		N/A			N/A	N/A		N/A	N/A			N/A					
Shedding (VP/single hamster genome copy)																																		
Feces Day 1															0,5							0,1												
Feces Day 3																									121,2									
Feces Day 6																																		
Feces Day 12																																		
Feces Day 21																									N/A	N/A	N/A	N/A						
Feces at necropsy																																		
Urine Day 1													15,8																0,6					
Urine Day 3													0,0																1,0					
Urine Day 6																																		
Urine Day 12																																		
Urine Day 21																							23,2		N/A	N/A	N/A	N/A						
Urine at necropsy																																		
Saliva Day 1									1,1																									
Saliva Day 3													14,3																					
Saliva Day 6																																		
Saliva Day 12																																		
Saliva Day 21																									N/A	N/A	N/A	N/A						
Saliva at necropsy																																		
Histopathological alterations (1=minimal, 2=slight, 3=moderate, 4=marked, 5=severe, P=present)																																		
Liver: Periportal infiltration, MNC*, multifocal	1		1							1		1					4				1			1									2	1
Liver: Lobular aggregates, MNC*, multifocal													1				3	1																
Heart: Left auricle, perivascular, MNC* infiltration, focal	1																																	
Kidneys: Papillary mineralization, (multi)focal					1	1			1						1			1						1				1	1				1	
Kidneys: Cortical mineralization, focal							1																											
Urinary bladder: Dilatation, diffuse (P = present)												P						P						P										
Prostate Interstitial, MNC* infiltrate, multifocal								1									1																	
Epididymides:																																		1
*Mononuclear cell infiltrates, mainly lymphocytes, scattered macrophages and plasma cells																																		
Blood hemathology																																		
Animal ID	1A	1B	1C	2A	2B	2C	3A	3B	3C	4A	4B	4C	5A	5B	5C	6A	6B	6C	7A	7B	7C	8A	8B	8C	9A	9B	9C	9D	10A	10B	11A	11B	11C	11D
B-TPK (x10e9/L)	964			774	777	781	1076		549		1046		1005		635	894	1080	972	759	696	566		760	903	686	809	798	693	798	773	1095	1132		352
B-Hb (g/L)	166		161	168	167	167	165		174		155		153		171	161	167	166	167	175	176	170	173	176	158	156	175	170	160	175	163	155		157
B-EVF (L/L)	0,52		0,5	0,53	0,53	0,53	0,52		0,53		0,49		0,49		0,53	0,5	0,53	0,51	0,53	0,54	0,54	0,52	0,54	0,52	0,49	0,49	0,54	0,53	0,51	0,54	0,5	0,49		0,49
B-LPK (x10e9/L)	6,9		3,6	7,2	6,4	4,7	3,4		5,3		5,8		7,4		7,6	4,1	8,5	3,5	9	4,1	5,5	4,3	5,4	4,4	5,9	4,7	5,6	5,8	5,1	6,8	6,1	7		4
B-Segm.kärn.neutrofiler (x10e9/L)	2		1	3,5	1	0,8	1,2		1,6		1,4		2,5		2,2	1,1	2,1	0,6	2,8	0,9	1,1	0,7	1,3	1	1,5	0,9	1,4	1,2	1,4	1,2	1,3	1,8		0,8
B-Eosinofiler (x10e9/L)	0,3		0,1	0,3	0,2	0,2	0,1		0,1		0,2		0,2		0,1	0,2	0,2	0,1	0,7	0,1	0,2		0,1	0,1	0,4	0,3	0,1	0,1	0,1	0,1	0,2	0,2		0,1
B-Lymfocyter (x10e9/L)	4,4		2,4	3,4	4,9	3,6	2		2,9		4		4,5		4,9	2,9	5,6	2,8	5,4	3	4	3,4	3,9	3,3	3,9	3,4	3,9	4,4	3,5	5,3	4,4	4,8		3,1
B-Monocyter (x10e9/L)	0,1		0,1	0,1	0,2	0,1	0,1		0,7		0,1		0,1		0,3	0,1	0,5	0,1	0,1	0,1	0,1		0,1	0,1	0,1	0,1	0,2	0,1	0,1	0,2	0,2	0,2		0,1
Serum biochemistry																																		
ALAT (µkat/L)	0,5	0,7	0,9	1,2	0,5	0,6	1,0	1,7	0,6	0,8	0,9	0,9	2,8	0,7	0,6	1,0	0,4	2,1	0,6	1,3	0,5	2,4	0,5	0,9	0,9	0,7	1,1	0,8	0,6	0,9	0,7	1,2	0,8	0,7
ASAT (µkat/L)	1,3	1,2	2,2	2,2	0,6	1,2	2,1	3,2	1,0	1,3	1,0	3,7	5,8	0,6	0,8	0,6	0,7	3,0	1,9	6,0	0,9	6,1	0,9	1,0	1,6	0,8	1,2	1,3	2,0	1,9	1,6	3,3	0,7	1,2
ALP (µkat/L)	2,7	2,3	1,3	1,9	2,1	2,1	2,0	1,5	2,6	1,7	2,5	2,4	2,0	2,4	1,5	1,5	2,5	1,8	1,8	2,0	2,1	1,4	2,6	2,6	1,8	2,1	1,9	1,3	2,5	1,8	2,0	2,3	2,7	1,6
Albumin (g/L)	33,3	33,3	35,0	32,5	30,4	31,0	33,9	33,8	32,5	37,3	32,6	30,7	32,8	33,8	34,4	33,3	32,2	32,6	33,8	31,4	32,4	35,8	31,7	33,8	31,9	32,4	31,5	30,8	30,4	34,7	33,9	30,4	32,8	31,0
Protein (g/L)	58,8	56,3	58,0	52,5	54,1	53,2	60,9	57,9	56,2	60,3	57,0	55,2	59,3	58,7	59,2	54,4	56,1	54,1	54,7	61,7	58,4	72,8	58,7	61,3	52,9	58,9	58,2	51,5	56,5	61,7	54,5	61,3	59,2	52,6
Kreatinin (umol/L)	16,2	15,4	13,2	12,5	17,1	17,4	12,6	11,3	16,0	16,9	17,2	15,3	12,8	18,1	15,9	16,7	19,4	8,0	6,0	4,8	12,1	3,9	11,8	7,9	7,9	11,6	9,5	8,3	9,9	3,4	7,8	8,1	13,5	7,4
Anti-adenovirus IgG	0,39	0,34	0,17	0,22	0,21	0,14	0,87	0,41	0,67	1,23	1,01	1,14	1,12	1,24	1,08	1,73	0,89	1,04	0,63	0,17	0,70	1,71	1,57	0,80	1,92	1,80	1,02	0,72	1,33	1,62	0,29	0,29	0,27	0,20
Hemolysis index												3,8								3,34		5,85									3,44			

Sample not recovered or not collected
